# A nanophotonic interferometer

**DOI:** 10.1088/1361-6528/acb443

**Published:** 2023-02-15

**Authors:** Abbas Ghaffari, Somayeh Kashani, Kevin Do, Keith Weninger, Robert Riehn

**Affiliations:** 1 Department of Physics, NC State University, Raleigh, NC 27695, United States of America

**Keywords:** zero mode waveguide, sub-wavelength aperture, interferometer

## Abstract

The transmission of light through sub-wavelength apertures (zero-mode waveguides, ZMW) in metal films is well-explored. It introduces both an amplitude modulation as well as a phase shift to the oscillating electromagnetic field. We propose a nanophotonic interferometer by bringing two ZMW (∼100 nm diameter) in proximity and monitoring the distribution of transmitted light in the back-focal plane of collecting microscope objective (1.3 N.A.). We demonstrate that both an asymmetry induced by the binding of a quantum dot in one of the two ZMW, as well as an asymmetry in ZMW diameter yield qualitatively similar transmission patterns. We find that the complex pattern can be quantified through a scalar measure of asymmetry along the symmetry axis of the aperture pair. In a combined experimental and computational exploration of detectors with differing ZMW diameters, we find that the scalar asymmetry is a monotonous function of the diameter difference of the two apertures, and that the scalar asymmetry measure is higher if the sample is slightly displaced from the focal plane of the collecting microscope objective. An optimization of the detector geometry determined that the maximum response is achieved at an aperture separation that is comparable to the wavelength on the exit side of the sensor. For small separations of apertures, on the order of a quarter of the wavelength and less, the signal is strongly polarization dependent, while for larger separations, on the order of the wavelength or larger, the signal becomes essentially polarization-independent.

## 1. Introduction

The detection of small quantities of biological molecules without fluorescence is a well-established goal, with the objective of achieving single-molecule detection at a concentration at micromolar concentration [1]. Classical interferometric methods can detect phase differences between separate beams of light with high sensitivity, and can be used to determine differences in refractive index with very high sensitivity [2]. If one is attempting to detect small quantities of materials, such as proteins or nucleic acids, in a nanofluidic setting using an interferometric method, one is confronted by the fact that the wavelength of light is often larger than the short optical path lengths that these liquid volumes provide for probing. That is a particular problem for interferometric methods since a sufficient phase difference between paths has to be accumulated over a short interaction distance. Other existing optical techniques that aim to detect minute quantities of biological analytes in small volumes solve this problem through the use of resonant photonic structures that slow the group velocity of light, such as in the spectral sensing of plasmon resonance of nanoparticles [3–5], resonant metal features in metal films [6, 7], or macroscopic whispering gallery mode resonators [8]. These techniques typically probe the intensity of scattered or transmitted light as a function of wavelength and require either a variable-wavelength source, or the knowledge that the nanostructure and incident light are stable over time while a single wavelength is being probed.

We aim here to remove the focus from the intensity and rather consider the direction of scattered light that is principally a function of the phase in an interferometer. The interferometer is formed by two sub-wavelength apertures in a metallic film that are spaced a few aperture diameters apart. The sub-wavelength apertures [9, 10], or nano-holes, through a metallic film are often termed zero mode waveguides (ZMW) when they are used for fluorescent detection of single molecules in biological contexts where the protein concentration is too high for conventional fluorescence microscopy [11]. Starting from Bethe’s description of transmission through an aperture in a perfectly conducting film [12, 13], later attention focused on the impact of surface plasmons on the transmission properties [14]. In particular, plasmons in arrays of sub-wavelength apertures lead to extraordinary transmission [15] that is tunable through the binding of biological molecules [16], and engineering of the plasmonic spectrum of a single aperture leads to directional transmission and emission from a single aperture [17].

The proposed interferometer is conceptually similar to an earlier design that was based on nanoslits [18] in the sense that it uses directional sensing of the transmitted light from a nanophotonic structure. However, we replace the nanoslits with ZMW because of the desired reduction in probe volume, and we will not utilize a moving detector or fiber pick-up as is usual in the field [19]. In order to allow for fast detection of the transmission pattern, we instead use an image sensor that detects the intensity in the back-focal plane of a high numerical aperture (N.A.) microscope objective which is used to collect the light transmitted by the ZMW pair [20]. In order to avoid heating the device/sensor at mW-scale laser power that might damage molecules in a biological sample, a 820 nm laser was used together with a gold film to reduce the absorption of light in the device.

We term our twin-ZMW device a near-field interferometer since the directional pattern of transmission of a twin ZMW can be described in terms of interference, and thus is a form of an interferometry. Because of the strong coupling between the electromagnetic field and the metal forming the ZMW, the field experiences a phase lag while traversing the apertures, leading to an increased interaction time between the wave and the lumen of the ZMW. In this paper, we demonstrate that introduction of a refractive index contrast between two ZMW through binding of a quantum dot (Q-Dot) in one of the two ZMW leads to an asymmetry in the back-focal plane pattern where any phase difference/direction of the transmitted light can be monitored. However, reasons other than a refractive index difference can also lead to phase difference of the light transmitted through the two ZMW, namely an asymmetry in the diameter of the apertures, or an asymmetry in the illumination. We use a diameter difference to develop of comprehensive physical understanding of the ZMW sensor. We anticipate that any physical description that successfully reflects the geometrical asymmetry can also account for an asymmetry in refractive index. In this publication we describe the instrument necessary to perform the proposed measurements, provide a basic characterization of the data obtained, and introduce a scalar measure of the asymmetry of the complex data. We then perform a basic optimization of the detector geometry and explore fundamental limitations of the device geometry [21].

## 2. Methods

### 2.1. Device fabrication and layout

**Figure 1.**
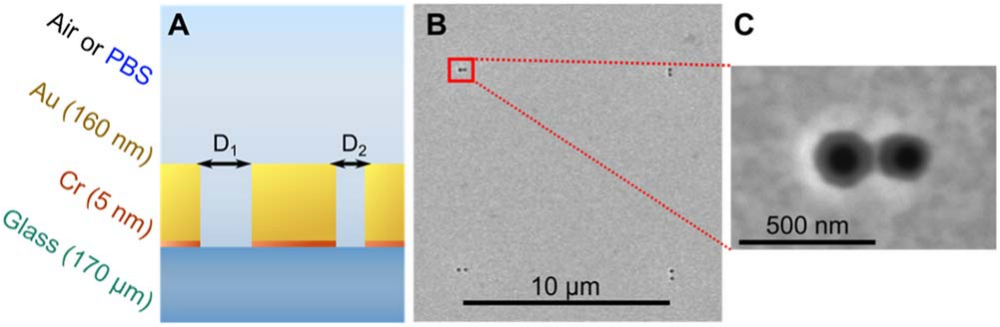
Structure of ZMW pair sensor. (A) Cross-section of device structure. (B) SEM of device region with four ZMW pair sensors. (C) Zoom of one pair in panel (B) with a 240 nm spacing, an average hole size of 105 nm, and a diameter difference of 15 nm.

Figure 1 illustrates the structure of the proposed nanophotonic interferometer. Our basic device consists of a ZMW pair through a gold film. The physics of the interference process is probed by variation of the diameter of the sub-wavelength apertures including symmetric and asymmetric pairs, the orientation of the axis connecting the two apertures, as well as the separation (center-to-center spacing). Figure 1(A) shows the schematic of an asymmetrical aperture pair that, depending on the study, was filled with air or phosphate-buffered saline (PBS) buffer solution. The 120–160 nm-thick gold film is supported by a 170 *μ*m-thick fused silica substrate with a 5–10 nm chromium adhesion layer in between. Metals were deposited using d.c. sputtering. ZMW are fabricated by focused ion beam milling using Ga^+^ ions. As illustrated in figure 1(B), ZMW pairs are laid out in a square array such that the distance between neighboring pairs is 10 *μ*m, sufficient to neglect interaction among pairs. Figure 1(C) provides a detailed view of one pair. Within each array, we provide hole pairs with different orientations (vertical/horizontal), hole separation (130–650 nm), and aperture diameter (90–130 nm). The dimensions were verified by scanning electron microscopy (SEM).

### 2.2. Experimental setup

**Figure 2.**
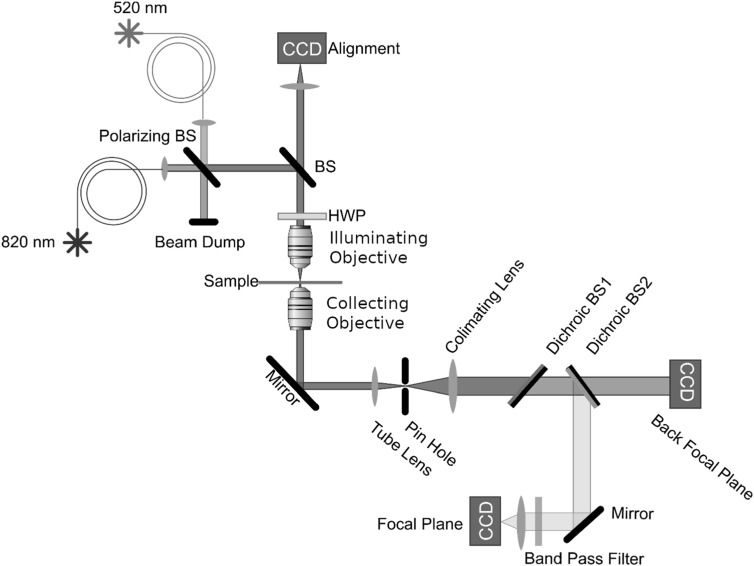
Schematic of optical setup. The setup is a microscope in transmission mode which enables monitoring of the direction of the transmitted light through sample from an area that is selected by a pinhole.

Our optical testing apparatus is configured as a transmission microscope as shown in figure 2. Fiber-coupled 520 and 820 nm lasers (Thorlabs, LP520-SF15 and LP830-SF30) are focused on the sample through a long-working distance microscope objective (Mitutoyo, 5×, effective N.A. = 0.04, w.d. = 34 mm) to form a diffraction-limited 10 *μ*m laser spot under normal incidence on the gold film. The N.A. and diameter were chosen such that the incoming beam can be approximated as a plane wave. A half-wave plate allows polarization control. ZMW transmission was collected under 820-nm illumination, while fluorescence was collected under illumination at 520 nm. An oil-immersion objective (Nikon, 100×, N.A. = 1.3, w.d. = 0.2 mm) collects the light transmitted by the ZMW pair and is imaged by a matched tube lens (Nikon) onto a 300 *μ*m pinhole. The pinhole selects a 3 *μ*m-wide area on the gold film, and the selected transmitted light is re-collimated by a single aspheric convex lens (*f* = 100 mm).

The green light is split off using a dichroic beam splitter (BS1, reflect ≤538 nm), and the Q-Dot fluorescence is separated from the infrared transmitted light using a dichroic beam splitter (BS2, reflecting ≤785 nm) and cleaned up using a bandpass filter (545–619 nm). An image of the ZMW pair in the fluorescence spectral window is focused onto the focal-plane CCD (Andor Luca) using a lens with 60 mm focal length. For confirming that the ZMW pair was at the focal plane, we removed the bandpass and imaged the ZMW pair directly using the focal-plane CCD using the small amount of reflected light. The infrared light transmitted by the dichroics BS1 and BS2 is collected by a second CCD (Thorlabs, DCC1545M) that we term the back-focal CCD.

In the manuscript we will detail that we can in general not guarante that optics are free of chromatic aberrations, and thus we apply the term ‘back-focal plane’ although it is not strictly applicable. We further even introduce an intentional defocus in many of the experiments, but still term this detector the back-focal CCD. Because of the spatial filter implemented by the combination of the near-field structure and pinhole, the pattern of light in the back focal aperture of the microscope objective is approximately the angular spectrum of the light transmitted by the near-field sensor. However, because we do not strictly enforce the focal condition on all conjugate planes, we cannot directly transform the back-focal-plane image into the angular spectrum of transmission. We thus present data in real space units as observed on the back-focal-plane CCD. For comparing the experiment to computational models, we transform the computed angular spectrum into real space measurement units as described in section 2.4.

### 2.3. Surface functionalization and materials

In order to tether a Q-Dot at the bottom of the ZMW, and also reduce the nonspecific adsorbtion, we performed chemical surface functionalization following the ideas of Roberto *et al* [22]. For measurements that tested the deflection under Q-Dot binding, the gold was passivated using a thiolated polyethylene glycol (mPEG-SH, MW 1000 Da, Laysan Bio, Inc.), and the glass interface at the bottom of the ZMW was functionalized using a biotin-terminated PEG-silane (Biotin-PEG-Silane, MW 3400 Da, Laysan Bio, Inc.). A fluidic system was formed by sealing the device with a polymer spacer (PARAFILM M) and a glass coverslip. We measured both the infrared transmission pattern as well as the fluorescence from the detector before and after exposure to Q-Dots (Q10133MP, Invitrogen, streptavidin conjugate, ∼25 nm diameter, maximum emission wavelength at 565 nm) while the device contained 100 mM PBS buffer.

### 2.4. Numerical simulations

**Figure 3.**
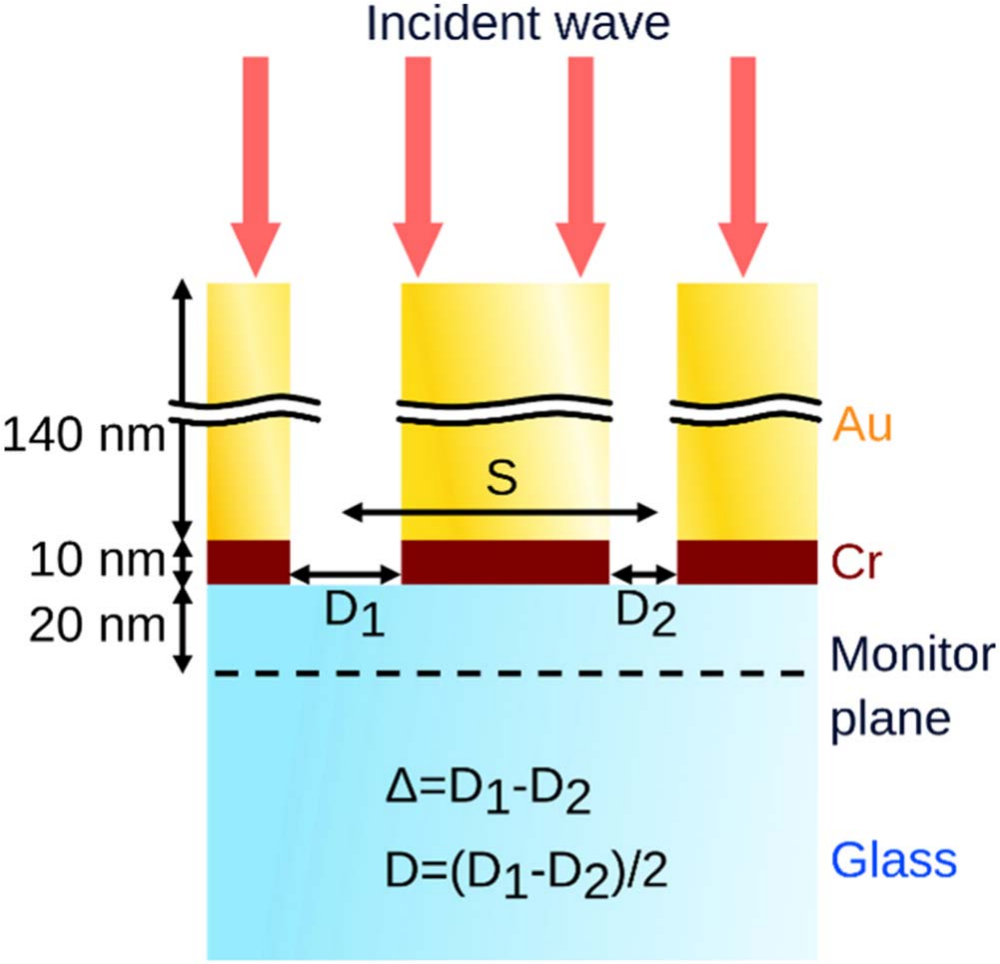
Simulation setup showing the dimensions and variable definitions. *S* is the separation, *D* is the mean diameter, and *Δ* is the diameter difference.

We used the finite-difference time-domain (FDTD) approach to simulate the spatial and directional transmission pattern of double sub-wavelength apertures as implemented in Lumerical FDTD (Ansys, 2021), a commercial software. The simulation domain was a three-dimensional box was spanned by a nonuniform mesh and surrounded by a perfectly matched layer. A pulsed Gaussian source at normal incidence illuminates the ZMW pair from the air side (top, *n* = 1.0) and the observation occurs on the glass side (bottom, *n* = 1.45). The schematic in figure 3 introduces the variables as they are used throughout this publication. A 6-parameter analytical fit to the experimental data published by Johnson and Christy was used for the refractive index of gold [23], and particular attention was paid to matching the imaginary component of the refractive index in the vicinity of 820 nm. The best agreement between experiment (160 nm thick gold film) and simulation was obtained for a metal stack with 140 nm of gold deposited on top of 10 nm of chromium. The small difference to the experimental gold thickness is either due to measurement uncertainty for the gold thickness, or a slight mismatch of the gold model due to the details of the gold deposition.

A plane monitor was located 20 nm below the chrome layer to collect the transmitted total fields for the duration of the simulation (200 fs). Complex-valued nearfield-to-farfield transformation was performed, and observable transmitted patterns were obtained using two different approaches. The first method was to use Fresnel diffraction to propagate the output of the FDTD simulation through the entire optical train using Zemax OpticStudio (Zemax, Ver. 22). As we will show, these results in general are a good representation of the experimental results for the back-focal-plane CCD, but due to the necessary resampling over an expanding and contracting beam combined with the limited resolution of the sampling we found speckle pattern formation could compromise the overall result, especially for the focal plane CCD or patterns with high spatial frequencies. For the focal-plane CCD, we instead truncated the angular spectrum resulting from the nearfield-to-farfield transformation to the N.A. of the collecting microscope objective, and then transformed back into a spatial pattern to obtain a scaled image that could be detected in the absence of any imaging aberrations. For some back-focal-plane images, we propagate the truncated angular spectrum after nearfield-to-farfield transformation along the optical axis before transforming into a spatial pattern and found good qualitative agreement in cases where the Fresnel propagation had yielded unstable results due to multiple mesh transitions between small and large surfaces.

## 3. Results and discussion

The discussion is organized in the following fashion. We first present a demonstration that single Q-dot nanoparticles can be detected by the twin-ZMW detector. We next qualitatively explore the patterns observed in both the back focal as well as the focal planes for sensors with asymmetry diameters. We then develop a single quantitative measure that can be used to characterize the sensor and show that the signal of that quantitative measure is maximized by a specific sensor geometry. We finally argue that for large hole separations the holes can be approximated as two independent sources that carry amplitude and phase information, while for small separations both apertures are strongly coupled if the incident polarization is aligned with the pair.

### 3.1. Quantum dot detection

**Figure 4.**
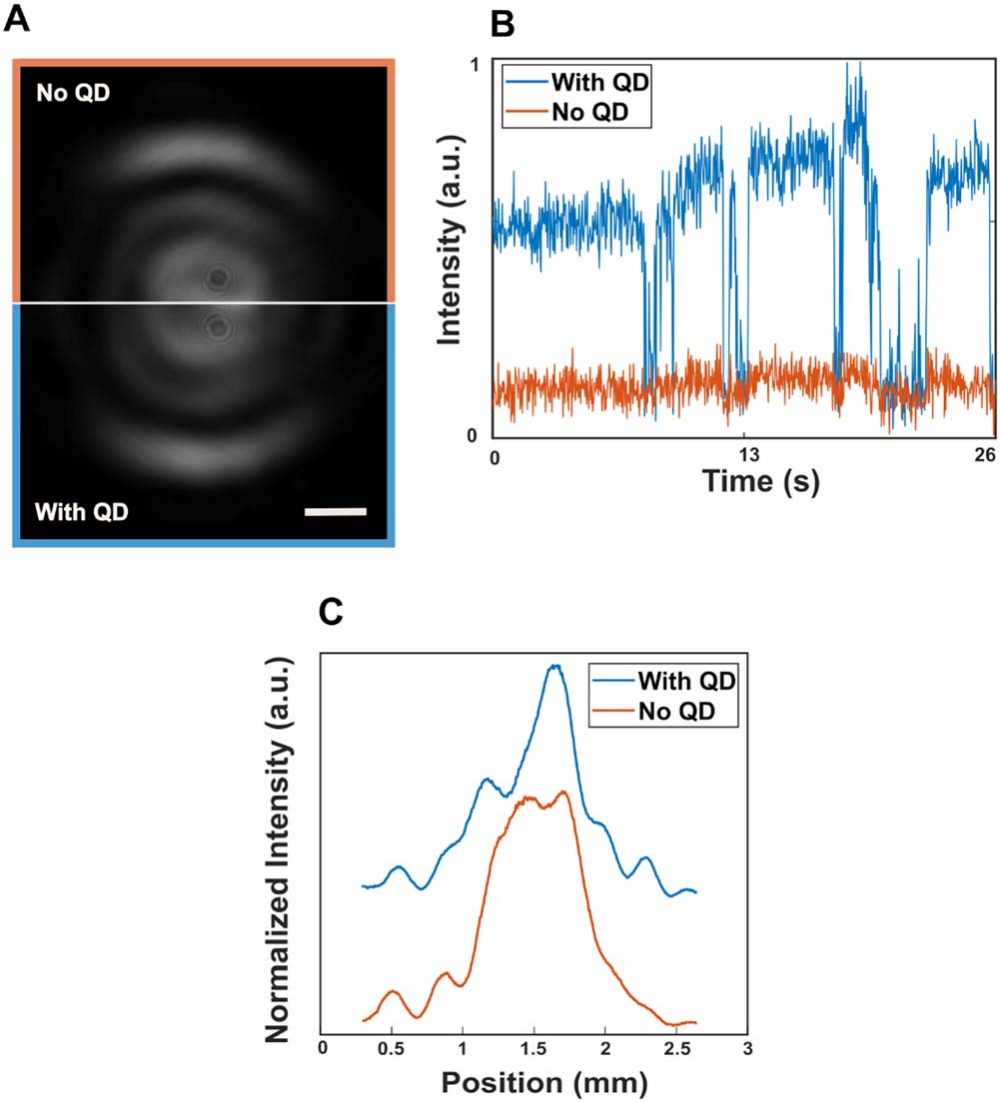
Detection of single Q-Dot by ZMW pair. (A) Back-focal-plane half images at 820 nm illumination. Red frame (top): No exposure to Q-Dot. Blue frame (bottom): Exposure to Q-Dot. Scale bar is 0.4 mm. (B) Time-resolved fluorescence intensities (blue with Q-Dot, red no Q-Dot) recorded on focal-plane CCD under 520 nm illumination. (C) Intensity distribution along the horizontal axis in panel (A) that connect two holes (blue with Q-Dot, red no Q-Dot).

The presence of a particle inside one of the apertures introduces a refractive index contrast that results in a phase difference between the apertures. Figure 4(A) presents the back-focal half images of a double aperture with average hole diameter of 150 nm and hole separation of 550 nm milled in a 130 nm gold film and illuminated by a laser of wavelength 820 nm before and after introduction of Q-Dots. The top half image (red frame) shows the reference before Q-Dot exposure, when holes are only occupied with buffer solution. The bottom half (blue frame) shows the transmission after Q-Dot exposure (Q-Dot concentration 20 nM). To verify presence of a Q-Dot in the detector we recorded a focal-plane image under 520 nm illumination (not shown) that indicates that only one of the two ZMW shows a fluorescence signal. To quantify the number of Q-Dots, we present the time-resolved fluorescence intensities for the two ZMW in figure 4(B). For the ‘dark’ ZMW, we observe a constant fluorescence background that is limited by the stability of our detector. For the ‘bright’ ZMW, the signal is elevated, and blinking events, that is switching between the elevated level and the background, are observed. The observation of blinking can be interpreted as the presence of a single Q-Dot [24–26], and the presence of a single Q-Dot in one of the two ZMW of the pair leads to a change/deflection in the far-field radiation pattern of the aperture pair. In order to quantify the differences of the two patterns presented in figure 4(A), we plot the intensity profile along symmetry (horizontal) axis in figure 4(C), and later apply a quantification that is described in the section 3.3.

### 3.2. Qualitative description of transmitted intensity

**Figure 5.**
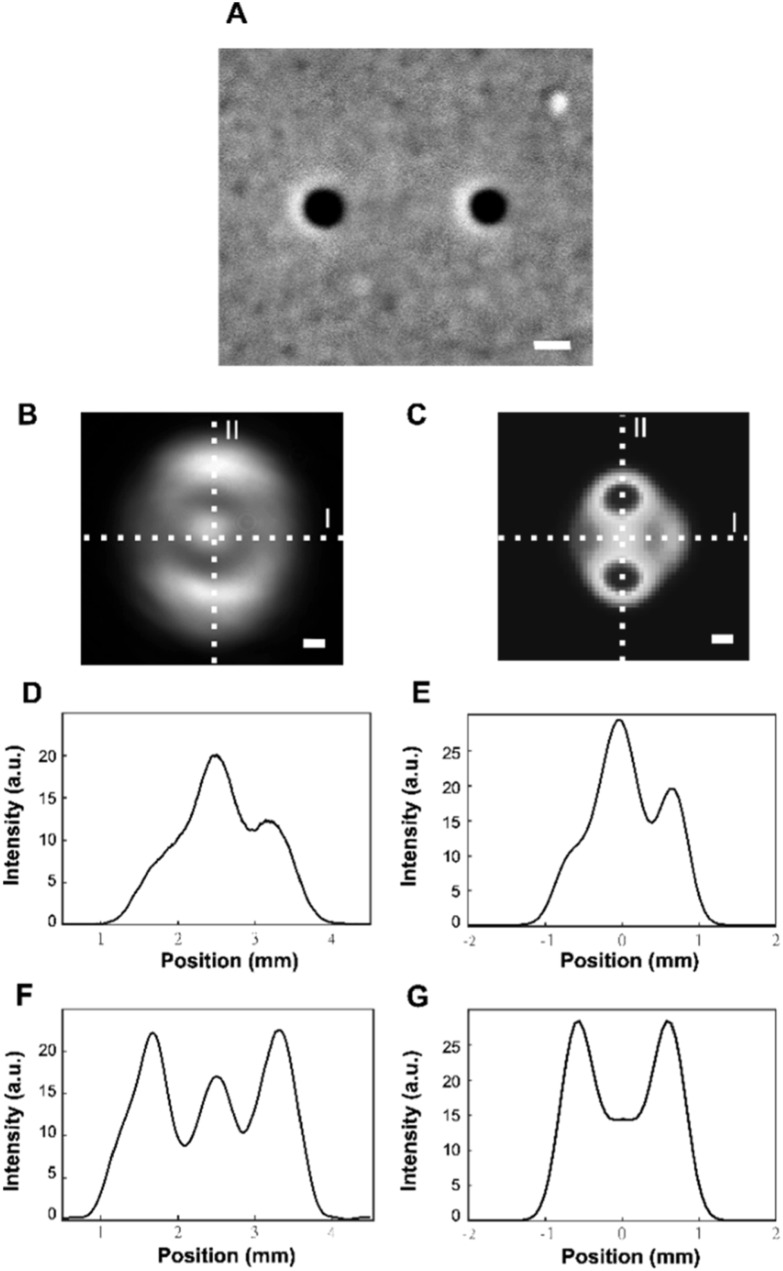
Transmitted intensity on back-focal CCD by pairs with 445 nm horizontal separation, mean hole diameter of 106 nm, and diameter difference of 12 nm with 820 nm horizontally-polarized illumination. (A) SEM image, scale bar 100 nm. (B) Experimental data, scale bar 0.3 mm. (C) Computed data. Scale bar 0.3 mm. (D) Experimental profile along horizontal cross section (labelled I in A). (E) Computed profile along horizontal cross section (labelled I in (B). (F) Experimental profile along vertical cross section (labelled II in A). G Computed profile along vertical cross section (labelled II in C).

Variation of Q-Dot binding location challenges systematic study as we show later. However, an asymmetry in ZMW size also creates a difference in phase. In order to describe the observed deflection in the transmitted pattern caused in presence of a Q-Dot within a ZMW, we systematically fabricate asymmetrical pairs (double ZMW of different diameters, as shown in figure 5(A)) and investigate the introduced phase difference/deflection in the transmitted intensity in the back-focal plane. In figures 5(B) and (C) we show experimental and computational back-focal plane intensity maps, respectively, of a horizontal ZMW pair with an average hole diameter of 106 nm, a hole diameter difference of 12 nm, and a hole spacing of 445 nm. Here, the thickness of the gold film is about 160 nm and ZMWs are filled with air. The polarization of the incident light is along the symmetry axis of the ZMW pair, i.e. the axis that connects the centers of the two apertures. The correspondence of measured and computed intensities is apparent. Note that the axial resolution of the computational result is less than the depth of field of the collecting microscope objective, that smoothing was applied to the computed image to suppress some speckle patterns, and thus some differences are to be expected. The pattern can be conceptualized as two major maxima along a line that runs perpendicular to the symmetry axis of the pair, and a ring-like structure along the axis of symmetry. In a later section we will show that this structure is robust under rotation of the polarization for large hole separations, but not for small separations. We prepared sections of the intensity profile along lines indicated in figures 5(B) and (C), and find that only the section along the (horizontal) symmetry axis of the ZMW pair carries information about the asymmetry.

**Figure 6.**
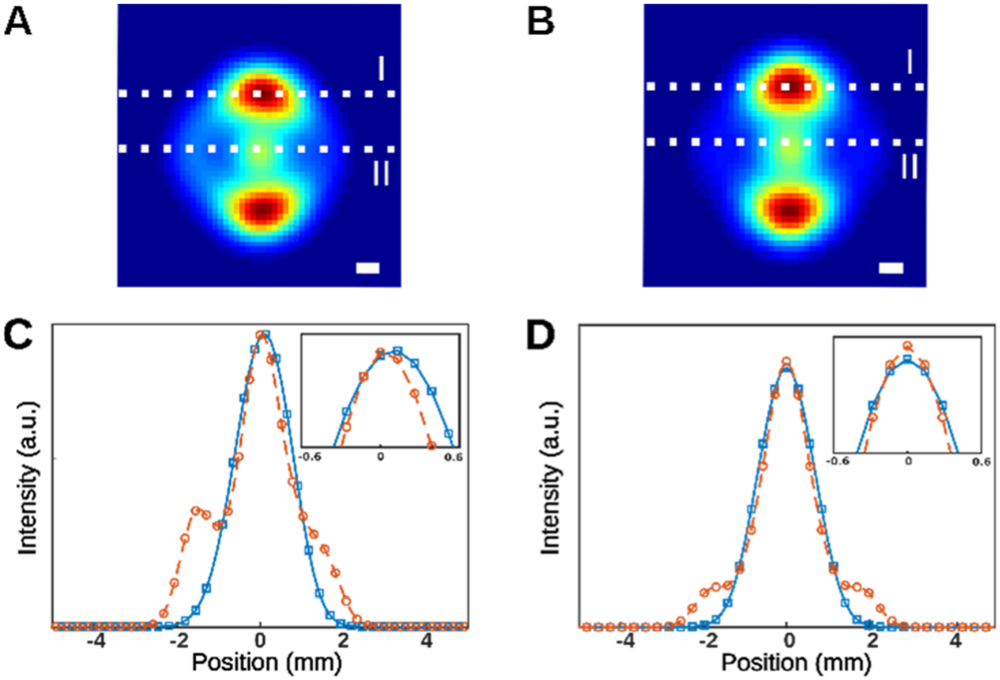
Simulations of transmitted intensity profile on back-focal plane CCD by ZMW pairs with 400 nm separation. (A) Asymmetric pair with ZMW diameters of 80 nm and 100 nm. Scale bar is 0.3 mm on CCD chip. (B) Symmetric pair with equal diameters of 90 nm. Scale bar is 0.3 mm on CCD chip. (C) Cross-sections for asymmetric pair. Blue solid line with squares is profile along section I in panel (A). Red dashed line with circles is section II in panel (A). (D) Cross-sections for the symmetric pair. The solid line with squares is profile along section I in panel (B). Red dashed line with circles is section II in panel (B).

It is at this point not clear whether this asymmetry of the intensity profile along the symmetry axis should be understood in terms of the different transmitted intensities through the individual holes, their phase difference, or whether the asymmetry stems from an illumination that does not sample the center of the incident Gaussian and thus carries directional information in itself. A simulation helps us answer this question since the optical axis is precisely known here, while misalignment is possible in the experiment. In figures 6(A) and (B) we compare the transmission through an asymmetric and a symmetric ZMW pair (separation 400 nm, mean diameter 90 nm, diameter difference 20 nm for the asymmetric pair). Sections along the symmetry axis in figures 6(A) and (B) (Red circles in figures 6(C) and (D)) show that the asymmetry in figure 5(C) is indeed due to the asymmetry of the pair. Figures 6(A) and (B) also contain strong evidence of a phase shift in the way that would be expected in a double-slit geometry. In particular, we demonstrate in figures 6(C) and (D) that the lateral position of the two maxima above and below the symmetry axis shift for asymmetric holes relative to the normal, while the position is not shifted for symmetric holes (Blue squares in figures 6(C) and (D) and insets therein). This lateral shift of the maxima is larger than the small shift that the central peak on the symmetry axis experiences.

**Figure 7.**
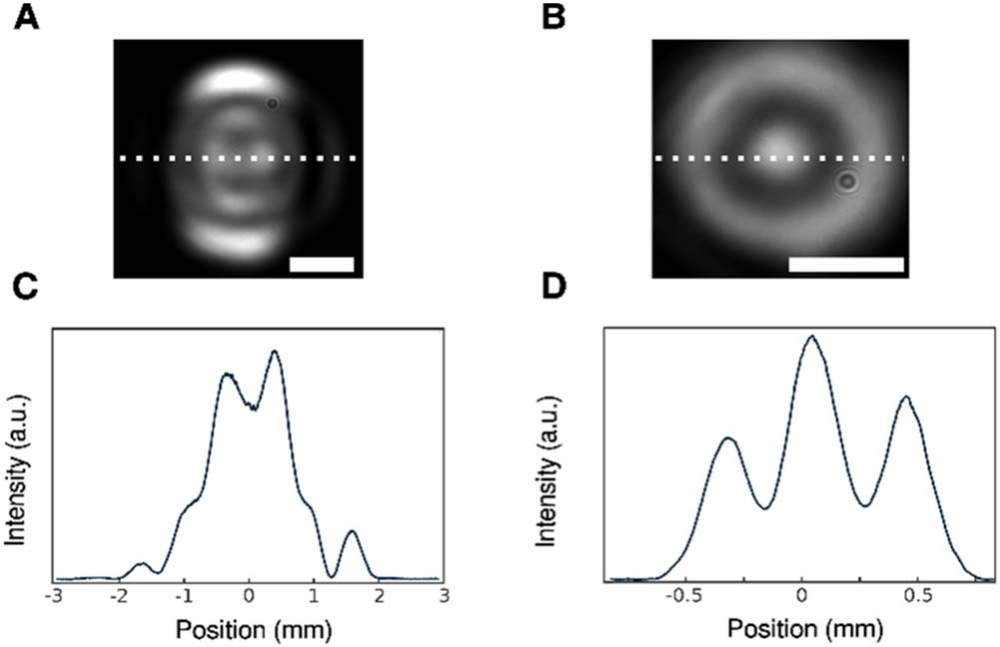
Patterns for large and small separations. (A) Back-focal profile from device with 620 nm spacing, mean diameter of 108 nm, and diameter difference of 13 nm. Scale bar is 1 mm. (B) Back-focal images of ZMW pair with 240 nm hole spacing, mean diameter of 105 nm, and diameter difference of 14 nm. Scale bar is 0.5 mm. (C) Section through A along the indicated line. (D) Section through B along the indicated line.

As the separation between the apertures is varied, the pattern evolves. We show this effect qualitatively in figure 7. For very large ZMW separations, diffraction beyond the first order becomes observable along the symmetry axis, and additional peaks emerge along the symmetry axis (figure 7(A) shows separation of 620 nm). Conversely, for very small hole spacing the pattern assumes near symmetry under rotation (figure 7(B) shows separation of 240 nm).

**Figure 8.**
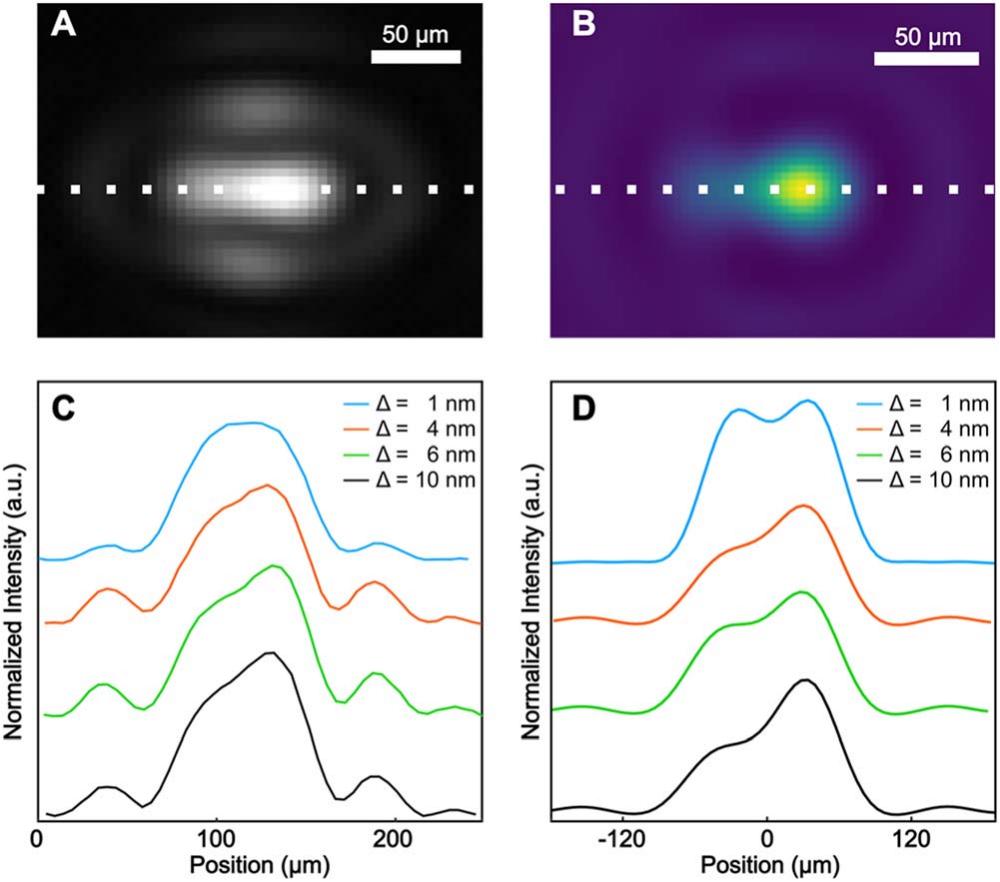
Focal plane images of ZMW pair with 650 nm hole spacing and an average hole diameter of 109 nm. (A) shows the experimental image for a diameter difference of 10 nm, while (B) shows the simulation for the same condition. (C) studies the intensity profile along the symmetry axis in the experimental data of panel A (indicated there as a dashed line) as well as diameter differences of 1 nm, 4 nm and 6 nm. (D) shows the intensity profile along the same axis in the simulated data of panel B and the other diameter differences.

An important consideration concerning the proposed measurement of the transmission direction is whether the proposed method yields any advantage over a straight-forward recording of the transmitted intensities of the two ZMW within a pair since the transmitted amplitude is doubtlessly also sensitive to the geometry of each ZMW as well as the refractive index within the lumen of each ZMW. To do so, we selected a ZMW pair with a design hole separation of 650 nm to enable easy resolution of the individual apertures on the focal-plane CCD after replacing BS1 and BS2 with mirrors, and removing the bandpass filter. In figure 8 we present experimental and computational results for ZMW pairs illuminated with light polarized parallel to the pair axis at 820 nm. Figures 8(A) and (B) show the observed and simulated image of transmission. On visual inspection, good agreement is achieved, and the asymmetry of the transmitted amplitudes is prominent.

### 3.3. Quantification of changes in transmission patterns

**Figure 9.**
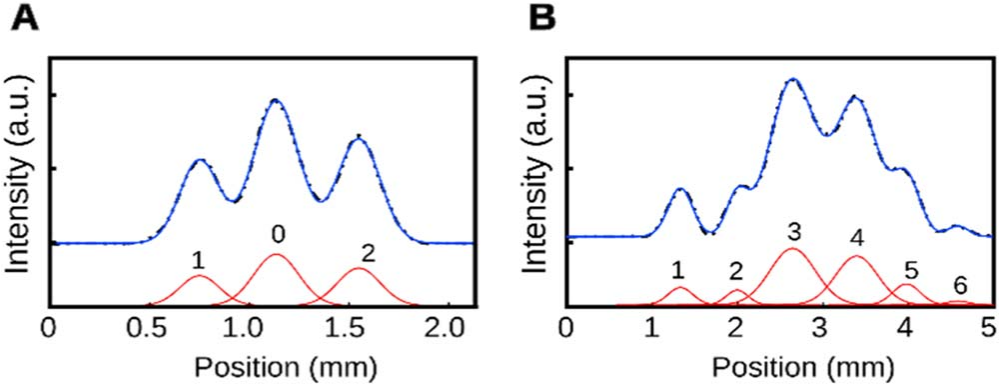
Illustration of fitting of sections along symmetry axis for small and large separations. (A) Back-focal profile from device with 240 nm spacing, mean diameter of 105 nm, and diameter difference of 14 nm. The scalar asymmetry is 0.14. (B) Back-focal profile from device with 640 nm spacing, mean diameter of 102 nm, and diameter difference of 6 nm. The scalar asymmetry is 0.18. Dashed black lines are experimental data, blue lines are the composite fit, and red lines are the individual Gaussians that constitute the fit.

In order to use the proposed ZMW pair structure as a sensor, we need a single scalar quantity to measure the difference in transmission (amplitude and phase) between the two holes. We will base this measure on the intensity along the symmetry axis of the ZMW. The measure has to be robust over a reasonably wide range of conditions, and in particular the emergence of a varying number of peaks. We fit a series of Gaussian peaks along the symmetry axis of the ZMW pair, where we record the areas under the fitted Gaussians as illustrated in figure 9. The scalar measure of ‘Asymmetry’ is then formed through:

\begin{eqnarray*}{\rm{Asymmetry}}=\displaystyle \frac{\displaystyle \sum {A}_{{\rm{odd}}}-\displaystyle \sum {A}_{{\rm{odd}}}}{\displaystyle \sum {A}_{{\rm{odd}}}+\displaystyle \sum {A}_{{\rm{odd}}}}.\end{eqnarray*}

Here A_odd_ and A_even_ are the areas under odd peak and even peak, respectively. Two distinct cases of application are possible. For traces that retain the central peak characteristic for small hole separations, the central peak is assigned index 0 which does not have a contribution in calculation of asymmetry. This is the case in figure 9(A), where we show a pair with a spacing of 240 nm. For large separations, the central peak was unimportant, and 0 was not assigned as an index. That is the case in figure 9(B), where we show a pair with a spacing of 640 nm with a scalar asymmetry of 0.18.

We now return to the quantification of the signal obtained through binding of a single Q-Dot in figure 4(C). We observe a change of the quantitative asymmetry change from 0.23 (no Q-Dot) to 0.39 (one Q-Dot), well beyond the uncertainty of the asymmetry measure for asymmetric sensors without Q-Dots which is at most on the order of 0.07. In order to examine the error rate of these sensors as binary detectors for presence of a Q-Dot, we compared both the back-focal CCD pattern and the fluorescence signal before and after exposure to Q-Dots. The fluorescence signal (change versus no change) was considered the ground truth, while the back-focal CCD pattern was considered the signal whose fidelity we test. Within a set of 10 sensors that reported a rise in fluorescence signal after Q-Dot exposure, 8 sensors showed Q-Dot detection through change in the transmitted pattern, while two did not show a deflection within the uncertainty stated above (true positive rate of 80%, false negative rate of 20%). Within a set of 8 sensors that reported no rise in the signal after Q-Dot exposure, we observed none with an asymmetry value beyond our threshold (false positive rate <12.5%, true negative rate >87.5%).

**Figure 10.**
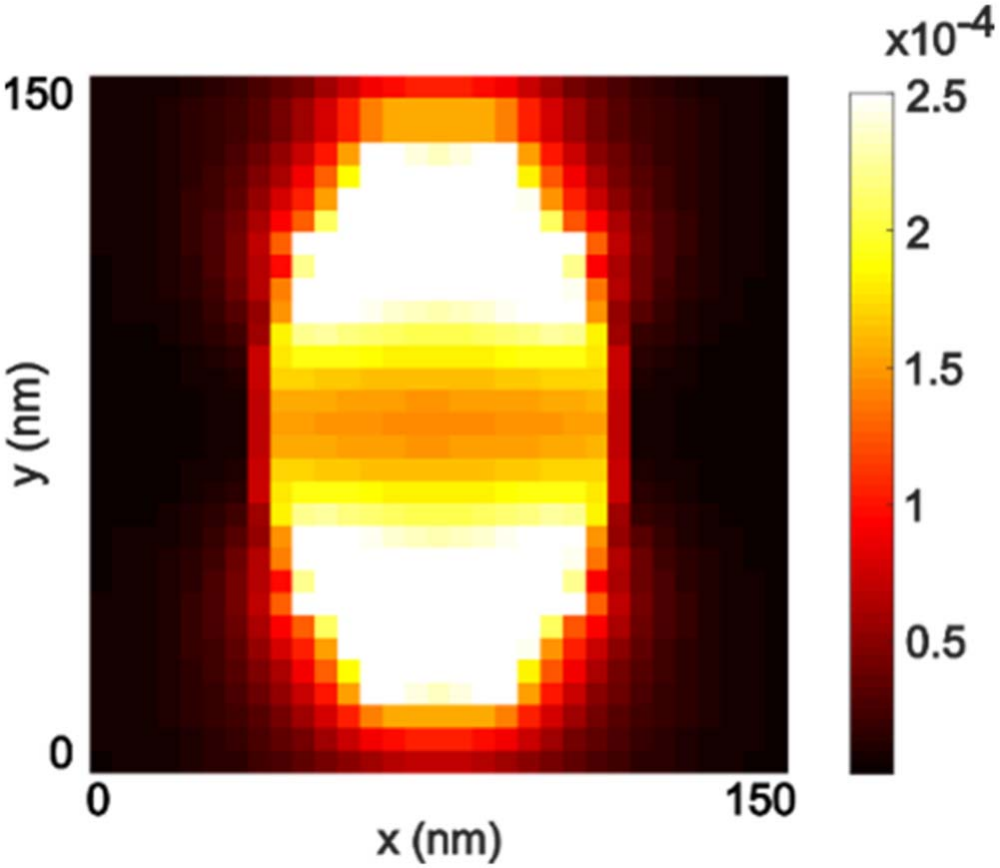
FDTD simulation of intensity distribution at the bottom of ZMW.

A comparison of detected and simulated asymmetries is not possible since the coupling between the electromagnetic fields and the nanoparticle is dependent on the electromagnetic intensity at the binding location of the Q-Dot. The intensity is highly inhomogeneous throughout across a ZMW (figure 10), and the interferometric asymmetry signal is anticipated to vary with the random binding location. In particular, we anticipate that a particle bound to the center of the ZMW would contribute less than an off-center bound particle.

**Figure 11.**
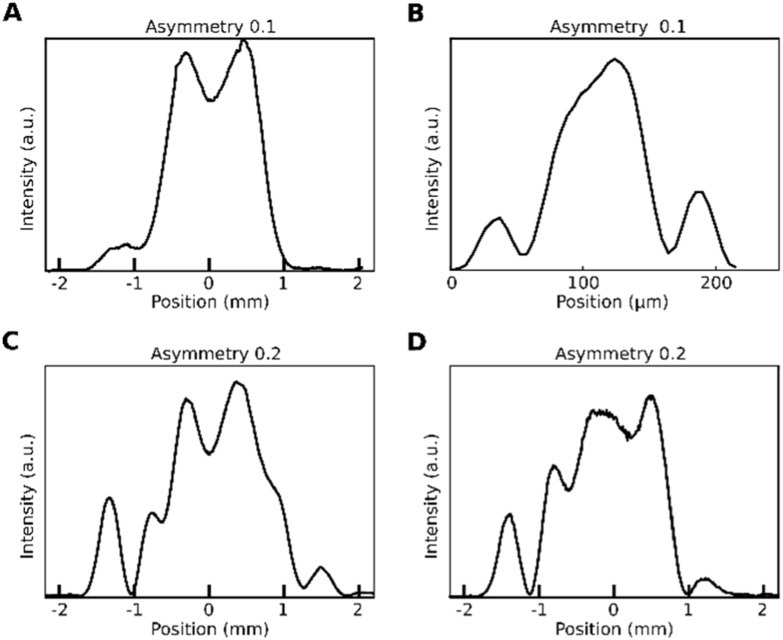
Evolution of axial profile of transmission pattern by a ZMW pair with separation of 650 nm, average hole diameter of 110 nm, and diameter difference of 10 nm for observation planes with numerical asymmetries indicated. (A) Back-focal CCD while sample is at focal point. (B) Focal CCD with best achievable focus. (C) Back-focal CCD while sample is displaced by +500 nm from focal point. (D) Back-focal CCD while sample is displaced by −500 nm from focal point.

**Figure 12.**
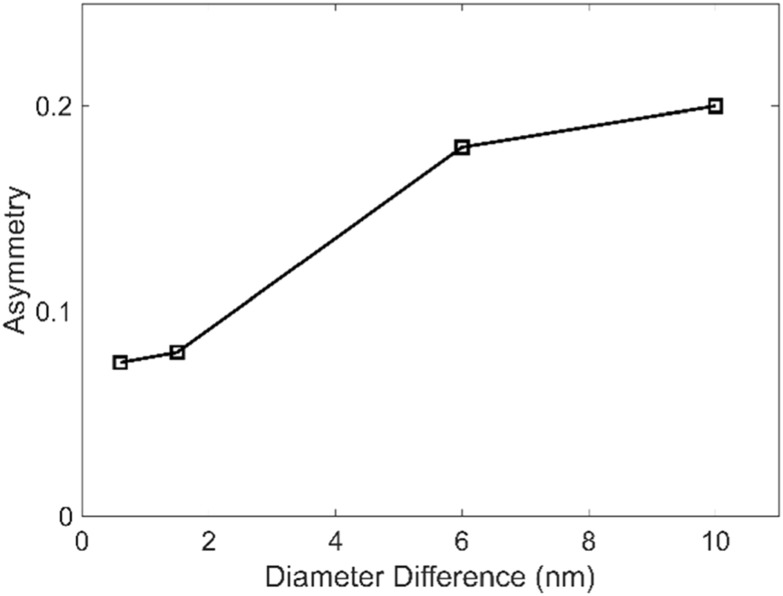
Asymmetry versus hole diameter for devices with an approximate hole separation of 650 nm and an approximate mean hole diameter of 111 nm measured on the back-focal CCD with a +500 nm displacement of the sample from the microscope objective focal plane.

In the preceding section we had mentioned the narrow depth of focus of the microscope objective and the possibility of recording the back-focal plane under a condition in which the object is not at the infinite conjugate of the microscope objective. We investigated whether this impacts the asymmetry measure by comparing the asymmetry in the back-focal CCD close to when the sample is in the focal plane (infinite conjugate), when the image is placed ±500 nm defocused from the infinite conjugate, and when the image is formed on the focal plane CCD at focus (figure 11). We find that the asymmetry is considerably larger for the defocused profiles on the back-focal CCD (both asymmetry of 0.2) than when the sample is in focal plane (asymmetry of 0.1), notably, the asymmetry in back-focal plane under defocus was also larger than the asymmetry on the focal-plane CCD in perfect focus. It thus appears that the back-focal CCD with the defocused sample is ideal for observing asymmetries of ZMW geometries, and we choose the +500 nm plane for the sample in the following unless indicated otherwise. In figure 12 we plot the scalar asymmetry signal as a function of difference in hole diameter while the mean hole diameter as well as the spacing between holes are held constant at 650 nm, the average mean hole diameter is 94 nm, and the diameter difference is varied between 1 and 10 nm. We find a monotonous function which reaches a scalar signal asymmetry of 20% at a diameter difference of 10 nm (diameter asymmetry of 10%). This indicates that this is a measure which is operationally feasible. This is a central result of this publication.

Quantitative measures of the transmitted profile other than the back-focal plane asymmetry are likely possible but are not as easily operable. For instance, in the preceding section we had described how the maxima above and below the symmetry axis performs lateral shifts due to a phase difference between the holes. However, that measure is not useful for the static shifts described in this paper since we do not have an intrinsic reference of the optical axis in the data. On the other hand, the scalar asymmetry measure along the symmetry axis evidently is sufficiently robust to any alignment uncertainties as different sensors on the chip are addressed.

**Figure 13.**
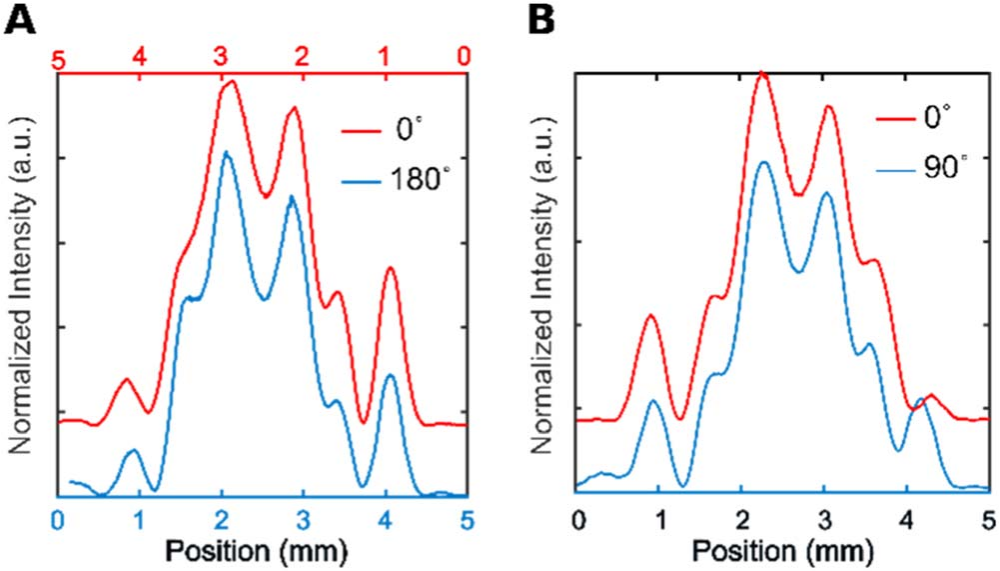
Test for asymmetries in transmission by collection optics. Panel (A) shows cross-section of back-focal CCD intensity as the same ZMW pair is rotated by 180° (separation of 640 nm, mean diameter of 110 nm, and diameter difference of 10 nm). Panel (B) shows show cross-section of back-focal CCD intensity of two different ZMW on the same wafer that have symmetry axes that are perpendicular to each other (separation of 640 nm, mean diameter 102 nm, diameter difference 6 nm). The polarization of the incident beam was parallel to the symmetry axis.

The optical system is of considerable complexity and thus any misalignment might cause asymmetries as well. Since the reported effect is an asymmetry along the symmetry axis, we investigated both horizontal inversions as well as rotation of the pair axis (figure 13). In figure 13(A) we present profile cross-sections with a polarization along the axis to study changes of the pattern under device rotation of 180°. We find asymmetry measures of 0.203 and 0.209 before and after rotation, respectively, and thus conclude that the measure is robust under inversion of the device axis. In the second test (figure 13(B)), we compared two separate ZMW pairs of orthogonal orientation on the same device that had otherwise identical design parameters. The incident polarization was rotated such that the polarization was kept along with pair symmetry axis. We found good agreement between the patterns before and after rotations, with scalar asymmetries of 0.17 and 0.18, respectively. Note that figure 13(A) (the 180° rotation) is a strong test of our ability to access focal planes repeatedly after remounting a device, while figure 13(B) (the 90° rotation) tests the repeatability of the fabrication and whether the waveplate introduces any artifacts.

### 3.4. Optimal designs for sensor performance

After establishing in figure 12 that the asymmetry in the back-focal plane pattern is a good scalar measure for the asymmetry of hole diameters, we proceeded to optimize the interferometer design. The central decisions for optimal sensor design to be made are the hole separation, the gold thickness, the polarization, and the mean diameter of the hole pair. The most highly constrained parameter is the gold thickness, since grains and pinholes associated with thin metal films degrade the sensor, while an excessive thickness leads to a diminished signal. We chose a thickness such that light leakage through the gold film was negligible under the 3-micrometer mask imposed by the pinhole located between the tube lens and the collimating lens in figure 2. The hole diameter is another constrained quantity, since large holes are less sensitive, while small holes have a high manufacturing uncertainty and low light throughput. Our chosen diameters were practical in the context of our CCD cameras. In this section we investigate the unconstrained parameters which are the spacing of the two ZMW and the polarization.

**Figure 14.**
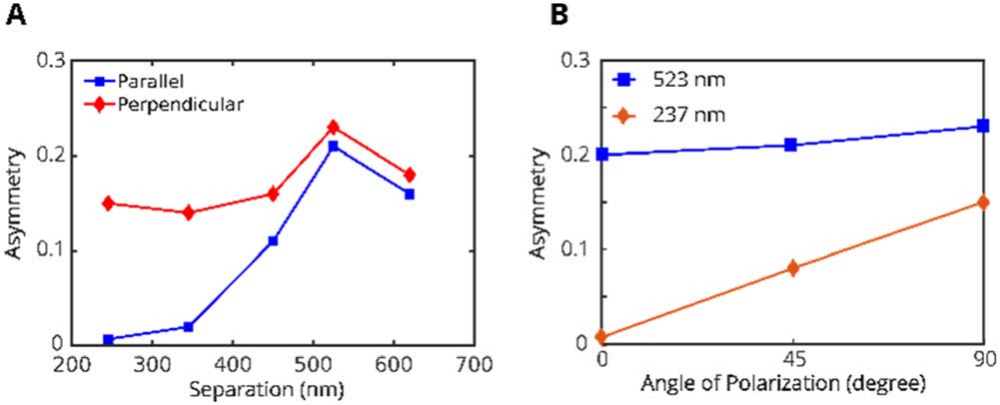
Measured asymmetry as function of hole separation and polarization for ZMW pairs with an approximate mean diameter of 110 nm and approximate diameter difference of 13 nm. (A) Experimentally measured asymmetries on the back-focal CCD. Blue squares are for polarization parallel to the pair axis, and red diamonds are for polarization perpendicular to the pair axis. (B) Comparison of the angular dependence of the scalar asymmetry measure for the large spacing (523 nm separation, 103 nm mean diameter, 14 nm diameter difference, blue squares) and small spacing (237 nm separation, 105 nm mean diameter, 14 nm diameter difference, red diamonds) devices.

In figure 14(A) we explore the impact of the ZMW separation in a series of experiments in which the mean diameter and diameter difference were held at 100 nm and 14 nm, respectively, while the design hole separation was varied between 250 and 650 nm and incident light was polarized along both possible axes. We find that the scalar asymmetry assumes a maximum at about 525 nm separation for either polarization, and even assumes the same value regardless of the polarization within the nanofabrication reproducibility. However, for smaller separations a polarization dependence emerges with a strongly reduced scalar asymmetry for polarization parallel to the symmetry axis than for polarization perpendicular to it.

We note that the optimum separation is on the order of the wavelength of 820 nm light in fused silica. This coincidence points us to consider a study by López-Tejeira *et al* [27], who investigated the transmitted total intensity of a finite one-dimensional chain under polarization along the chain direction as a function of the number of holes and wavelength for a fixed hole separation. Those authors had focused on hole separations on the order of the wavelength and found that a maximum in the transmission occurs at a wavelength marginally larger than the wavelength itself. This condition is met at about ∼560 nm separation, which is close to the maximum response of the scalar asymmetry.

The impact of the polarization on the scalar asymmetry is explored in figure 14(B). For hole separations of about one wavelength (523 nm), the rotation of the polarization from parallel to perpendicular relative to the symmetry axis leads to a ∼10% increase of the asymmetry measure, while for a separation of approximately half a wavelength (237 nm), the increase was more than 10-fold. These observations are consistent with a picture in which large separations are approximately described by independent transmission of light through the two ZMW, and the pattern is then formed through scalar diffraction. In contrast, for the smaller separations a joint plasmonic wave is efficiently excited in the gold film surrounding both holes when the polarization is parallel to the axis of the pair. In that case, the correlation between the field in both ZMW removes any directional asymmetry of the transmitted wave. However, if the polarization is perpendicular to the symmetry axis, the directional asymmetry measured in the back-focal plane is recovered since the joint plasmonic wave is not launched as efficiently.

**Figure 15.**
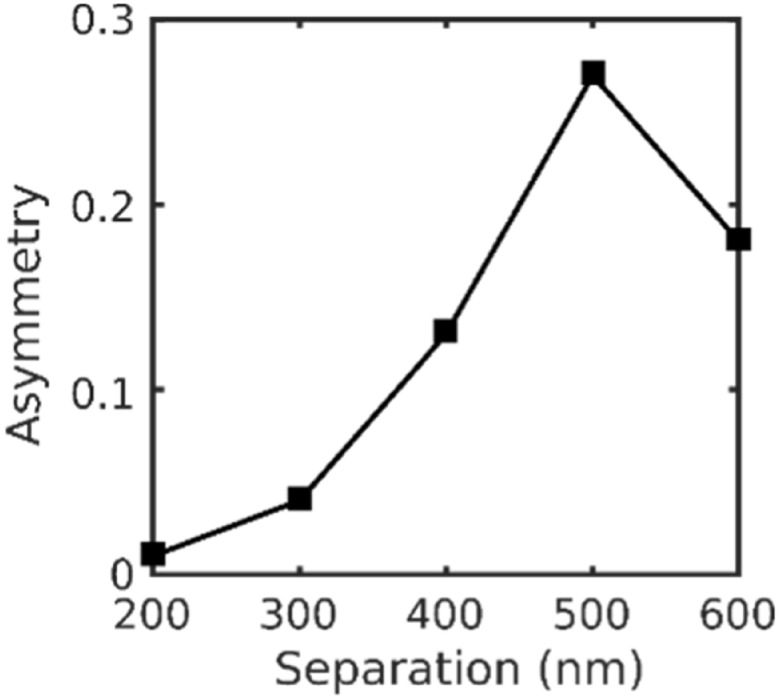
Computed asymmetries as function of hole separation and polarization for ZMW pairs with an approximate mean diameter of 110 nm, approximate diameter difference of 13 nm, and polarization parallel to the symmetry axis.

We conclude this section by presenting supporting simulations of the scalar asymmetry as a function of separation under parallel incident illumination that use an angular spectrum propagator to approximate the imaging condition in the back-focal plane with defocus (figure 15). We find a relationship similar to the one presented in figure 14(A), both in shape with a maximum at 550 nm separation as well as the order of magnitude of the scalar asymmetry measure. This indicates that the FDTD simulations capture the physics of the proposed interferometer including plasmonic effects well.

## 4. Conclusions

We have proposed a nanophotonic interferometer comprising two ZMWs embedded in a metallic film, and showed that the pair functions to detect single Q-Dot particles as well as asymmetries in the geometry of the two ZMWs by considering the directional distribution of the transmitted light. We identified a signature linked to the phase of the transmitted wave and introduced a scalar asymmetry to quantify the differences between complex transmission patterns. We optimized the detector geometry with a ZMW separation on the order wavelength of light on the exit side. At that separation, the detector shows approximately the same sensitivity for all polarization directions. Choosing a hole separation on the order of half the wavelength on the exit side with polarization parallel to the symmetry axis led to a strong reduction in the transmitted intensity while decreasing the sensitivity of the scalar asymmetry measure at the same time. We anticipate that improved nanophotonic interferometer that controls the binding location may also be used to probe the presence of biological macromolecules proteins in solution either through direct time-dependent observation, or measures analogous to dynamic light scattering or fluorescence correlation techniques while maintaining attoliter probe volumes and concentrations typical even for abundant proteins [28].

## Data Availability

The data that support the findings of this study are available upon reasonable request from the authors.
